# Severe COVID-19 Illness and α1-Antitrypsin Deficiency: COVID-AATD Study

**DOI:** 10.3390/biomedicines11020516

**Published:** 2023-02-10

**Authors:** Juan Luis Rodríguez Hermosa, Gianna Vargas Centanaro, María Estela González Castro, Marc Miravitlles, Lourdes Lázaro-Asegurado, Beatriz María Jiménez-Rodríguez, Rosanel Amaro Rodríguez, Rosaly Moreno Méndez, María Torres-Duran, José María Hernández-Pérez, Ana María Humanes-Navarro, Myriam Calle Rubio

**Affiliations:** 1 Pulmonology Department, Research Institute of Hospital Clínico San Carlos (IdISSC), 28040 Madrid, Spain; 2 Department of Medicine, Faculty of Medicine, Complutense University of Madrid, 28040 Madrid, Spain; 3 Pneumology Department, Hospital Universitario Torrecardenas, 04009 Almería, Spain; 4 Pneumology Department, Hospital Universitari Vall d’Hebron, Vall d’Hebron Institut de Recerca (VHIR), Vall d’Hebron Barcelona Hospital Campus, 08035 Barcelona, Spain; 5 Pneumology Department, Complejo Asistencial Universitario de Burgos, 09006 Burgos, Spain; 6 Pneumology Department, Hospital Virgen de las Nieves, 18014 Granada, Spain; 7 Pneumology Department, Hospital Clinic, 08036 Barcelona, Spain; 8 Pneumology Department, Hospital La Plana, 12002 Castellon, Spain; 9 Pneumology Department, Hospital Álvaro Cunqueiro, NeumoVigo I+i Research Group, IIS Galicia Sur, 36213 Vigo, Spain; 10 Pneumology Department, Hospital Universitario Nuestra Señora de La Candelaria, 38010 Santa Cruz de Tenerife, Spain; 11 Preventive Medicine Clinical Management Unit, Research Institute of Hospital Clínico San Carlos (IdISSC), 28040 Madrid, Spain

**Keywords:** SARS-CoV-2 infection, severe COVID-19, alpha-1 antitrypsin deficiency, genetic mutations

## Abstract

Background: Epidemiologic studies have reported that the geographical distribution of the prevalence of allelic variants of serine protein inhibitor-A1 (SERPINA1) and severe cases of COVID-19 were similar. Methods: A multicenter, cross-sectional, observational study to evaluate the frequency of alpha-1 antitrypsin deficiency (AATD) in patients with COVID-19 and whether it was associated with having suffered severe COVID-19. Results: 2022 patients who had laboratory-confirmed SARS-CoV-2 infection. Mutations associated with AATD were more frequent in severe COVID versus non-severe (23% vs. 18.8%, *p* = 0.022). The frequency of Pi*Z was 37.8/1000 in severe COVID versus 17.5/1000 in non-severe, *p* = 0.001. Having an A1AT level below 116 was more frequent in severe COVID versus non-severe (29.5% vs. 23.1, *p* = 0.003). Factors associated with a higher likelihood of severe COVID-19 were being male, older, smoking, age-associated comorbidities, and having an A1AT level below 116 mg/dL [OR 1.398, *p* = 0.003], and a variant of the SERPINA1 gene that could affect A1AT protein [OR 1.294, *p* = 0.022]. Conclusions: These observations suggest that patients with AATD should be considered at a higher risk of developing severe COVID-19. Further studies are needed on the role of A1AT in the prognosis of SARS-CoV-2 infection and its possible therapeutic role.

## 1. Introduction

Severe acute respiratory syndrome coronavirus 2 (SARS-CoV-2) has resulted in a pandemic, with more than 6 million deaths [1]. There are remarkably different infection and mortality rates for SARS-CoV-2 between different countries [2]. Moreover, there are remarkably interindividual differences in the clinical severity of coronavirus disease 2019 (COVID-19) that cannot be completely explained by environmental factors, comorbidities, and age-related fragility [3]. On the basis of these observations and the susceptibility of hosts, it could be argued that genetic differences among populations, ethnicities, and individuals may contribute to the different epidemiological and clinical manifestations of COVID-19 [4]. Recent studies have investigated genetic susceptibility to SARS-CoV-2 and reported that approximately 20% of life-threatening COVID-19 cases are associated with genetic errors and gene loci, most of which are involved in immune signaling pathways [5].

In-hospital COVID-19 patient, studies have described a proinflammatory syndrome with a disproportionately high rate of progression to acute respiratory distress syndrome [2]. Recent data indicates that the COVID-19 cytokinemia is distinct in critical care presentations, showing marked differences in the balance between proinflammatory and anti-inflammatory cytokines and a blunted alpha-1 antitrypsin (A1AT) acute phase response. Cytokine ratios, such as high IL-6:A1AT levels, are related to worse prognosis in COVID-19 patients [2].

Alpha-1 antitrypsin deficiency (AATD) is the most common inherited disorder in adults; it is often under-diagnosed [6] and characterized by reduced plasma levels or the abnormal functioning of A1AT, a human blood serine protease inhibitor, which is encoded by the serine protein inhibitor-A1 (SERPINA1) gene. Recent studies confirmed a correlation between the COVID-19 pandemic and the prevalence of AATD in the same geographical areas [7].

A1AT is a tissue protector, as well as an antiviral and anti-inflammatory molecule. Indeed, A1AT has several biological functions that may antagonize SARS-CoV-2 infection and pathophysiologic processes resulting in cellular entry. Recent studies have demonstrated that A1AT is an inhibitor of SARS-CoV-2 infection and two of the most important proteases in the pathophysiology of COVID-19: transmembrane serine protease 2 and the disintegrin and metalloproteinase 17, as was well as an inhibitor of inflammatory molecules, such as IL-8, TNF-α, and neutrophil elastase [8,9]. Other potential A1AT protective mechanisms of action are the inhibitory effect on thrombin and delayed thrombus formation [10] and decreased oxidative stress, inflammation, and cell wall deterioration [11].

Therefore, we focused on the possible role of AATD as a risk factor for severe COVID-19 progression. A poor prognosis for COVID-19 patients may be related to A1AT levels. In our study, we examined the presence of genetic mutations associated with AATD and A1AT levels in patients who had suffered a SARS-CoV-2 infection in order to assess whether AATD was associated with having suffered severe COVID-19.

## 2. Materials and Methods

COVID-AATD is a multicenter, cross-sectional, observational study conducted from 1 May 2021 to 1 September 2022. The sample population was adults who had laboratory confirmed SARS-CoV-2 infection and were treated by a pneumology department. Participants were enrolled consecutively at 9 centers in the inpatient ward or in follow-up consultation after discharge. There were no exclusion criteria, except for patients’ or families’ explicit refusal to participate. The study was performed according to the Declaration of Helsinki and its amendments. All patients gave written informed consent. The study was approved by the Research Ethics Committee at Hospital Clínico San Carlos, Madrid, Spain (internal code 20/809-E). The personal data of the patients was kept under strict confidentiality in compliance with the provisions of Spanish Organic Law 3/2018, of December 5, on the Protection of Personal Data and Guarantee of Digital Rights (LOPDGDD) and its development regulations, and in accordance with the provisions of Regulation (EU) 2016/679 of the European Parliament and of the Council of 27 April 2016, regarding the protection of natural persons with regard to the processing of personal data and the free circulation of these data.

A retrospective review was performed through the analysis of electronic medical records where SARS-CoV-2 infection clinical data were collected. Patients were defined as suffering severe COVID-19 if they had been treated with high-flow nasal cannula (HFNC) oxygenation, non-invasive ventilation therapy, or were admitted to the intensive care unit at any stage of the disease according to the WHO Clinical Progression Scale [12], or if they died as a result of COVID-19.

The data collected during the only visit were concurrent. The information collected was clinical data (demographic data, smoking status, comorbidities). Allele-specific genotyping testing was carried out in all patients using the Progenika A1AT Genotyping Test. The test allows the identification of the 14 most frequent deficiency variants of the SERPINA1 gene: PI*S, PI*Z, PI*I, PI*Mprocida, PI*Mmalton, PI*Siiyama, PI*Q0granite falls, PI*Q0west, PI*Q0bellingham, PI*F, PI*Plowell, PI*Q0mattawa, PI*Q0clayton, and PI*Mheerlen. SERPINA1 gene sequencing was performed in the cases where none of the 14 mutations were found and the A1AT serum level was <60 mg/dL. The test is CE marked and United States Food and Drug Administration approved. The test is intended for use with genomic DNA extracted from human whole blood samples collected in K3-ethylenediaminetetraacetic acid (EDTA) tubes or as dried blood spots (DBS), or from human buccal swab samples [13]. The biological samples related to the study were numbered with a code to guarantee the confidentiality of the sample and the associated clinical data. There were no data in the database that could be used to identify patients. The patients signed a written informed consent authorizing the genetic study to be carried out according to Spanish legislation.

In the clinical stability phase, serum A1AT levels were analyzed using nephelometric and C-reactive protein (CRP) in plasma as a potential confounder by the immunonephelometry method. Although the lower limit of normal A1AT by nephelometry is 90 mg/dL, the use of a higher than normal cut-off value was established as a threshold value to study the possible presence of a deficient allele. The variability of A1AT levels has been described for different AATD genotypes and how it may be influenced by increased systemic inflammation [14].

### Statistical Analysis

Descriptive statistics are reported as mean (standard deviation [SD]) or median (interquartile range [IQR]). Differences between the non-severe and severe COVID-19 groups were analyzed for statistical significance using the chi-square or Fisher’s exact test for categorical variables and the two-sample *t*-test or Wilcoxon rank sum test for continuous variables, as applicable. Adjustment variables (patient characteristics, genotyping test, and serum A1AT levels) with a *p*-value < 0.05 in the univariate analysis were included in the simple logistic regression analysis. Statistical significance was assumed as *p* < 0.05. All analyses were performed with Stata software version 17 (Stata Corp LLC, College Station, TX, USA). The study size was determined by the number of patients referred to the follow-up clinic in a pneumology department during the enrolment period.

## 3. Results

### 3.1. Characteristics of the Study Population

In total, 2022 patients were included in the analysis. Table 1 describes the sociodemographic and clinical characteristics of the enrolled patients. An amount of 43.2% had severe COVID-19 infection and six (0.3%) deaths occurred. The mean (SD) age of the overall COVID-19 cohort was 60.3 ± 14 years; 59.9% were men and 45.6% of patients were current or former smokers. Comorbidities were common in the study population.

A1AT serum levels were available in 1691 (83.6%) cases, with a mean value of 132.1 (28.8) mg/dL. There were 390 (19.9%) carrying frequent mutations (S or Z), and 14 (0.7%) carrying rare alleles. In total, 67 samples were not processed due to the poor quality of the sample or due to errors recording the identification code on the web. The prevalence of the frequent allele combinations in this selected population was as follows: MS 16.3%, MZ 2.1%, SS 1.1%, SZ 0.3%, and ZZ 0.2%. Considered globally, 2.5% were Z carriers and 17.6% S carriers.

### 3.2. Characteristics According to the Presence of Genetic Mutations Associated with AATD

Patients with variants of the SERPINA1 gene that could affect A1AT protein activity or expression were older than patients without mutations (mean [SD] age: 61.8 [14.1] versus 60 [13.9] years; *p* = 0.021) and current smokers were more prevalent (7.9% versus 5.3%; *p* = 0.004). There were no differences in respiratory or non-respiratory comorbidities, Table 2. The frequency of severe COVID was also higher in patients positive for A1AT genotyping testing (48.8% vs. 42.4%; *p* = 0.022). A1AT serum levels were significantly lower in patients with mutations associated with AATD (106.3 [24] versus 138.8 [25.8]; *p* < 0.001).

### 3.3. Characteristics According to A1AT Levels

**Figure 1:**
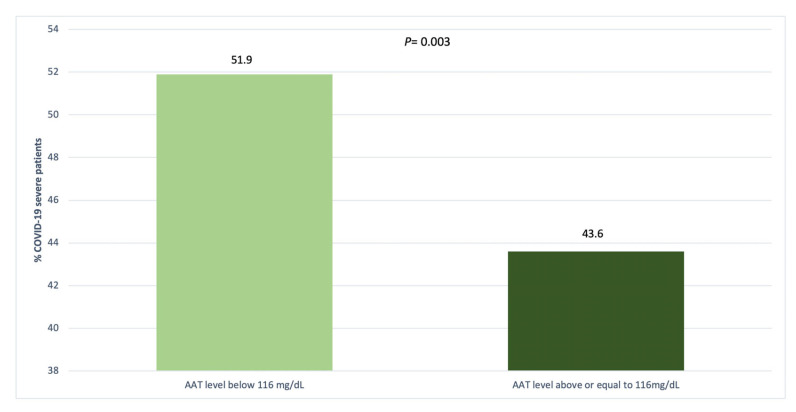
Distribution of COVID-19 severity by serum AAT levels.

There were 440 (26%) patients with A1AT serum levels below 116 mg/dL, Table 3. The frequency of severe COVID was higher in patients with A1AT serum levels below 116 mg/dL compared with those above or equal to 116 mg/dL (51.9% versus 43.9%, *p* = 0.003), Figure 1.

### 3.4. Characteristics According to COVID-19 Severity

**Figure 2:**
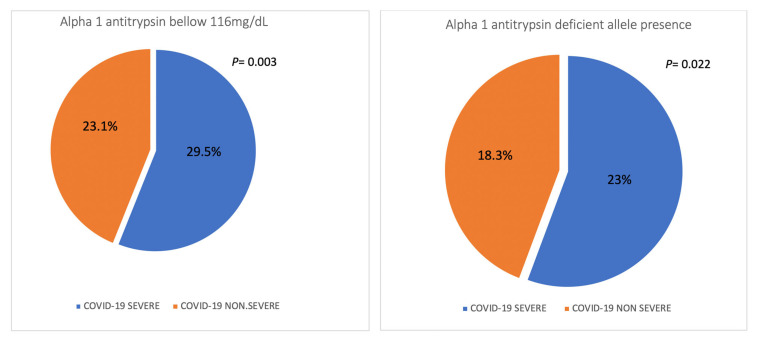
Distribution of the severity of COVID-19 in population with AAT levels < 116 mg/dL and in population with deficiency-related mutations.

There were 872 (43.2) patients defined as suffering severe COVID-19. Cases with severe COVID were older than patients with non-severe COVID (mean [SD] age: 62.8 [12.8] versus 58.9 [14.7] years; *p* < 0.001) and being male was more frequent (67.4% versus 54.3%, *p* < 0.001), Table 4. Having A1AT levels below 116 was more frequent in cases with severe COVID versus non-severe COVID (29.5% versus 23.1, *p* = 0.003). Cases carrying mutations associated with AATD were more frequent in severe COVID versus non-severe COVID (23% versus 18.8%, *p* = 0.022), Figure 2.

### 3.5. Factors Related to COVID-19 Severity

In the simple logistic regression analysis, the factors associated with a greater likelihood of having suffered severe COVID-19 were being older, being a male, being a former smoker, having cardiovascular comorbidities and a history of cancer, having A1AT levels below 116 mg/dL [OR 1.398 (CI95%: 1.124–1.739), *p* = 0.003], and having a mutation associated with AATD [OR 1.294 (CI95%: 1.038–1.612), *p* = 0.022], Table 5.

## 4. Discussion

This multicenter observational study investigates the association between AATD and the severity of COVID-19 in patients with a SARS-CoV-2 infection that were treated by pneumology departments in Spain. This analysis demonstrates that, having mutations, variants of the SERPINA1 gene that could affect A1AT protein activity or expression and that having decreased A1AT levels was significantly associated with a higher likelihood of suffering from a severe COVID-19 case. This is consistent with data that suggested that AATD might explain the high COVID-19 mortality in countries with a high AATD prevalence. During the COVID-19 pandemic, several epidemiologic studies have reported that the geographical distributions of the prevalence of SERPINA1 allelic variants and severe cases of COVID-19 were similar, although confounding factors should be considered in these analyses, such as the different control measures established by governments, SARS-CoV-2 vaccination, socioeconomic status, and population health [3,7,15]. Other observational studies in patients with AATD also found a higher frequency of SARS-CoV-2 infection and a higher risk for symptomatic SARS-CoV-2 infection in patients with severe AATD with lung disease [16,17]. Recently, an EARCO ERS Clinical Research Collaboration analysis that investigated the impact of COVID-19 on patients with severe AATD (PiZZ, PiSZ, or rare variants with an equivalent serum A1AT level < 60 mg/dL) [18] showed that while a poor outcome was more frequent in PiZZ compared with PiSZ, this did not reach statistical significance; non-respiratory comorbidities were more strongly associated with a poor outcome than genotype, baseline FEV1, or oxygen saturation. However, it should be noted that in this cohort of patients with AATD, although 88% were diagnosed with COVID-19 with a positive PCR, only 31% required hospitalization. In addition, an analysis of a community-based cohort with > 500,000 participants that assessed the association between AATD and COVID-19 in the United Kingdom Biobank showed that the most common and mild AATD genotypes were not associated with increased SARS-CoV-2 infection rates or increased SARS-CoV-2 fatalities, although it must be noted that there were very few cases of severe AATD in this study [19]. In our population of patients with SARS-CoV-2 pneumonia, the frequency of Pi*S was 176/1000 and Pi*Z 25/1000. These figures are high in relation to the estimated prevalence in Spain [20], with a mean SZ prevalence of 278/1000, Pi*Z 17/1000, and Pi*S 104/1000. This higher frequency of mutations related to severe impairment (ZZ, SZ) found in our cohort could support our hypothesis that a poor prognosis for COVID-19 patients may be related to the presence of genetic mutations associated with AATD. A large proportion of patients in our cohort required supportive therapies and intensive care for COVID-19, which could be explained by the fact that patients with more severe COVID-19 are usually referred to the pneumology follow-up clinic because they are at higher risk of developing complications [21]. Indeed, our data showed that cases carrying mutations associated with AATD were more frequent in severe COVID versus non-severe COVID (23% vs. 18.8%, *p* = 0.022). The frequency of Pi*Z was 37.8/1000 in severe COVID versus 17.5/1000 in non-severe COVID, *p* = 0.001. The presence of genetic mutations associated with AATD was found to be a predictor factor associated with a higher likelihood of suffering a severe COVID-19 case [OR 1.294 (CI95%: 1.038–1.612), *p* = 0.022], which was consistent with studies that confirmed a correlation between the frequency of Pi*Z and Pi*S alleles and mortality rates due to COVID-19 [7]. Furthermore, recent studies have investigated genetic susceptibility to SARS-CoV-2 and reported that approximately 20% of life-threatening COVID-19 cases were associated with genetic errors and gene loci, most of which are involved in two immune signaling pathways [5,22]. Thus, we could hypothesize that upon exposure to the same virus, while some individuals show asymptomatic or mild illness, plausibly due to effective immune reactions, severe COVID-19 patients may reflect dysfunctional immune reactions that lead to increased lung injury.

Regarding information on risk factors for the development of SARS-CoV-2 pneumonia and a severe disease course, our study supports many of the findings from previous reports indicating that the epidemiology of COVID-19 shows a diverse pattern across people who are different in age, sex, ethnicity, and particularly among those with pre-existing medical conditions [3,23,24,25]. In our cohort, being male, being older, having a history of smoking, and having age-associated comorbidities significantly contributes to the severity of acute COVID-19. However, it should be noted that in our analysis, patients with the presence of genetic mutations associated with AATD or A1AT levels below 116 mg/dL do not have a higher prevalence of hypertension, diabetes, heart disease, chronic kidney disease, or chronic obstructive pulmonary disease. However, there are other potential factors, such as the dominant COVID strain at the time of infection, as this is not an assessment that is performed in daily clinical practice, or vaccination status against SARS-CoV-2; however, vaccination coverage in adults in Spain was very high with more than 85% are vaccinated.

Several studies have focused on the possibility of shared pathogenic pathways between AATD and SARS-CoV-2 infection. Indeed, A1AT has several biological functions that may antagonize SARS-CoV-2 infection and pathophysiological processes. Alpha-1-antitrypsin is a tissue protector with antiviral and anti-inflammatory properties [26,27]. The main function of A1AT is inactivating proteolytic enzymes [28], which are released in pulmonary tissue. Furthermore, a protective role for A1AT has been described for several viral infections. A1AT levels may be relevant to the development of viral diseases as rhinovirus infection, human immunodeficiency virus, hepatitis B and C, and complications [29,30,31].

The relationship between a worse prognosis of SARS-CoV-2 infection and lower levels of AAT could be explained by the possible protective role of A1AT against COVID-19. A1AT reduces transmembrane serine protease 2 activity [27], protection against acute lung injury [32], and strong anti-inflammatory properties [10]. In relation to the potential A1AT protective mechanisms of action, our analysis showed that having A1AT levels below 116 was more frequent in cases with severe COVID versus non-severe COVID (29.5% versus 23.1, *p* = 0.003), and the presence of A1AT levels below 116mg/dL was identified as a predictor factor associated with a higher likelihood of suffering a severe COVID-19 case, which was consistent with studies that demonstrated the COVID-19 cytokinemia is distinct from that of other types of pneumonia. In these studies, the production and sialylation of A1AT are increased in COVID-19, but this anti-inflammatory response is overwhelmed in severe illness, with the IL-6: A1AT ratio being markedly higher in patients requiring ICU admission. In critically unwell patients with COVID-19, increases in IL-6: A1AT predicted a prolonged ICU stay and mortality, whereas improvement in IL-6:A1AT was associated with clinical resolution [2]. In this regard, supplementation of the acute A1AT response with exogenous A1AT may merit consideration, as it has been shown to modulate the production and activity of the key proinflammatory cytokines described in [28,33] while preserving the production of IL-10 [34]. Indeed, it has recently been reported that abrupt cessation of A1AT augmentation therapy for patients with AATD resulted in marked increases in levels of these specific proinflammatory cytokines, a loss of IL-10, and subsequent progression to respiratory failure [35].

Our study has several limitations. First, COVID-AATD is a cross-sectional, observational study in patients treated by pneumology departments and this may carry some bias. Patients with more severe COVID-19 are controlled mainly by pulmonologists, which may result in overestimates since it is more likely the most unwell patients are selected. On the other hand, most cases were included at the follow-up consultation after discharge, and very few were during hospitalization for COVID-19, which results in underestimated fatal COVID cases, despite the fact that the sample size is quite large and stratified by COVID infection severity. Second, the multiplex system studies the 14 most frequent mutations that include more than 99% of the deficient variants observed in the world. Therefore, the identification of Pi*M is achieved through exclusion, since the absence of any of these 14 alleles suggests with more than 99% probability that it is an M. However, when none of the 14 mutations were found and the A1AT serum level was <60 mg/dL, SERPINA1 gene sequencing was performed, which did not occur in our study. Third, although the lower limit of normal A1AT by nephelometry is 90 mg/dL, we established an above-normal cut-off value in our analysis on the basis that deficient mutations can be detected above this level and may also be influenced by increased systemic inflammation [36]. Consequently, the use of an above-normal cut-off value could be argued as a threshold value to screen for the possible presence of a deficient allele. Fourth, in the logistic regression analysis, other variables are not considered such as vaccines and specific therapies that could impact association estimates.

## 5. Conclusions

Our study identifies the presence of mutations associated with A1AT and that have A1AT levels below 116 as predictors associated with an increased likelihood of severe COVID-19. These observations suggest that patients with AATD should be considered at a higher risk of developing severe COVID-19. These findings highlight the need for further studies on the role of the A1AT in the pathogenesis and prognosis of SARS-CoV-2 infection and a potential therapeutic role.

## Figures and Tables

**Table 1:** Characteristics of the study population.

Enrolled Patients	n = 2022
Age, years, median (IQR)	61.2 (51–71)
Gender (male), n %	59.9
▪ Smoking status, (%)	
Current smoker	5.9
Former smoker	39.7
Never smoked	54.4
▪ IPA, median (IQR)	25 (12–40)
Body mass index, kg/m^2^, median (IQR)	29.2 (26.1–33)
Pulmonary comorbidity, n (%)	427 (21.1)
▪ COPD, n (%)	122 (6)
▪ Asthma, n (%)	186 (9.2)
▪ AOS, n (%)	151 (7.5)
▪ ILD, n (%)	23 (1.1)
Home oxygen therapy, n (%)	25 (1.2)
Comorbidities	
▪ Diabetes mellitus, n (%)	385 (19.1)
▪ Hypertension, n (%)	795 (39.4)
▪ Dyslipemia, n (%)	677 (33.5)
▪ Coronary artery disease, n (%)	226 (11.2)
▪ Nephropatia, n (%)	90 (4.5)
▪ Hepatopathy, n (%)	80 (4)
▪ Immunosuppression, n (%)	63 (3.1)
▪ History of cancer, n (%)	147 (7.3)
Bilateral pneumonia, n (%)	1533 (76.3)
Inpatient, n (%)	1592 (78.8)
Hospitalization day, median (IQR)	9 (3–17)
ICU/UCIR, n (%)	507 (25.1)
High flow oxygen or NIV/CPAP, n (%)	872 (43.2)
Deaths, n (%)	6 (0.3)
A1AT mg/dL, median (IQR)	129 (116–148)
▪ ≥116, (%)	74
▪ <116 y ≥ 60, (%)	25.6
▪ <60, (%)	0.4
CRP level, m (SD)	0.90 (0.29–4.42)
Genotyping Test, n (%)	
▪ Absence mutations	1551 (79.3)
▪ MI	6 (0.3)
▪ MS	318 (16.3)
▪ MZ	41 (2.1)
▪ MM malton	2 (0.1)
▪ MP lowell	5 (0.3)
▪ MM procida	1 (0.1)
▪ SS	22 (1.1)
▪ SZ	5 (0.3)
▪ ZZ	4 (0.2)

Abbreviations: IPA: Pack-years; COPD: chronic obstructive pulmonary disease, ILD: diffuse interstitial lung disease, AOS: sleep apnea syndrome, ICU: intensive care unit, UCRI: intermediate respiratory care unit; NIV/CPAP: non-invasive ventilation/continuous positive airway pressure; A1AT: alpha-1 antitrypsin; CRP: C-reactive protein.

**Table 2:** Characteristics of enrolled patients according A1AT genotyping test.

Enrolled Patients with Genotyping Test n = 1955	Absence Mutations n = 1551 (79.3%)	Presence Mutations n = 404 (20.7%)	*p*
Age, years, m (SD)	60 (13.9)	61.8 (14.1)	0.021
Gender (male), n %	921 (59.4)	255 (63.1)	0.176
▪ Smoking status, n (%)			
Current smoker	82 (5.3)	32 (7.9)	0.044
Former smoker	596 (38.7)	173 (43.3)	0.099
▪ IPA, median (IQR)	22.7 (10–40)	25 (15–40)	0.039
Body mass index, kg/m^2^, median (IQR)	29.2 (26.1–33)	29.39 (26–33.3)	0.618
▪ Pulmonary comorbidity, n (%)	333 (21.5)	84 (20.8)	0.785
COPD, n (%)	91 (5.9)	27 (6.7)	0.534
Asthma, n (%)	144 (9.3)	35 (8.7)	0.702
ILD, n (%)	17 (1.1)	4 (1)	1.000
Home oxygen therapy, n (%)	16 (1)	9 (2.2)	0.056
▪ Comorbidities, n (%)			
Diabetes mellitus	291 (18.8)	84 (20.9)	0.333
Hypertension	611 (39.4)	165 (40.9)	0.578
Dyslipemia	495 (32)	159 (39.5)	0.005
Coronary artery disease	179 (11.6)	45 (11.2)	0.839
Nephropatia	69 (4.5)	19 (4.7)	0.789
Hepatopathy	66 (4.3)	14 (3.5)	0.461
Immunosuppression	41 (2.6)	18 (4.5)	0.057
History of cancer	105 (6.8)	36 (8.9)	0.136
Bilateral pneumonia, n (%)	1165 (75.6)	328 (81.8)	0.009
Inpatient, n (%)	1211 (78,1)	342 (84,9)	0.003
Hospitalization day, m (SD)	8 (3–16)	11 (4–20)	<0.001
ICU/UCIR, n (%)	368 (23.7)	120 (29.8)	0.013
High flow oxygen or NIV/CPAP, n (%)	649 (41.9)	193 (48)	0.028
Severe COVID-19, n (%)	656 (42.4)	196 (48.8)	0.022
Deaths, n (%)	4 (0.3)	2 (0.5)	0.610
A1AT mg/dL, m (SD)	138.8 (25.8)	106.3 (24)	<0.001
▪ ≥116, n (%)	1153 (86.2)	91 (26.6)	<0.001
▪ <116, n (%)	184 (13.8)	251 (73.4)	

Abbreviations: IPA: pack-years; COPD: chronic obstructive pulmonary disease, ILD: diffuse interstitial lung disease, AOS: sleep apnea syndrome, ICU: intensive care unit, UCRI: intermediate respiratory care unit; NIV/CPAP: non-invasive ventilation/continuous positive airway pressure; A1AT: alpha-1 antitrypsin; CRP: C-reactive protein.

**Table 3:** Characteristics of patients, according A1AT level (≥116 mg/dL versus <116 mg/dL).

Enrolled Patients with A1AT Level n = 1691	A1AT ≥ 116 mg/dL n = 1251 (74%)	A1AT < 116 mg/dL n = 440 (26%)	*p*
Age, years, m (SD)	61 (14)	60.2 (13.2)	0.288
Gender (male), n %	744 (59.5)	279 (63.4)	0.151
▪ Smoking status, n (%)			
Current smoker	62 (5)	33 (7.5)	0.047
Former smoker	488 (39.2)	185 (42)	0.289
▪ IPA, median (IQR)	25 (11.4–40)	25 (15–40)	0.464
Body mass index, kg/m^2^, median (IQR)	29.3 (26.2–33.2)	29.6 (26.2–33.2)	0.455
Pulmonary comorbidity, n (%)	268 (21.4)	91 (20.7)	0.744
▪ COPD, n (%)	78 (6.2)	30 (6.8)	0.670
▪ Asthma, n (%)	117 (9.4)	35 (8)	0.380
▪ ILD, n (%)	10 (0.8)	6 (1.4)	0.389
Home oxygen therapy, n (%)	15 (1.2)	10 (2.3)	0.109
Comorbidities, n (%)			
▪ Diabetes_mellitus	251 (20.1)	85 (19.3)	0.731
▪ Hypertension	535 (42.8)	158 (35.9)	0.011
▪ Dyslipemia	435 (34.9)	160 (36.4)	0.569
▪ Coronary artery disease	159 (12.7)	38 (8.7)	0.022
▪ Nephropatia	56 (4.5)	16 (3.6)	0.449
▪ Hepatopathy	55 (4.1)	17 (3.9)	0.622
▪ Immunosuppression	28 (2.2)	12 (2.7)	0.561
▪ History of cancer	91 (7.3)	27 (6.1)	0.418
Bilateral pneumonia, n (%)	926 (74.4)	362 (83)	<0.001
Inpatient, n (%)	973 (77.8)	372 (84.5)	0.003
Hospitalization day, m (SD)	8 (3–16.2)	11 (5–19.7)	<0.001
ICU/UCIR, n (%)	296 (23.7)	135 (30.7)	0.004
High flow oxygen or NIV/CPAP, n (%)	539 (43.2)	224 (51)	0.005
Deaths, n (%)	4 (0.3)	2 (0.5)	0.654
Genotyping Test, n (%)			
▪ Absence mutations	1153 (92.7)	184 (42.3)	
▪ MI	2 (0.2)	4 (0.9)	
▪ MS	87 (7)	78 (40.9)	
▪ MZ	0	37 (8.5)	
▪ MM malton	0	2 (0.5)	
▪ MP lowell	2 (0.2)	3 (0.7)	
▪ MM procida	0	1 (0.2)	
▪ SS	0	18 (4.1)	
▪ SZ	0	4 (0.9)	
▪ ZZ	0	4 (0.9)	
A1AT mg/dL, m (SD)	143.4 (23.6)	99.8 (13.8)	<0.001
CRP_nivel, m (SD)	0.95 (0.29–5)	1 (0.20–6.25)	0.436

Abbreviations: IPA: pack-years; COPD: chronic obstructive pulmonary disease, ILD: diffuse interstitial lung disease, AOS: sleep apnea syndrome, ICU: intensive care unit, UCRI: intermediate respiratory care unit; NIV/CPAP: non-invasive ventilation/continuous positive airway pressure; A1AT: alpha-1 antitrypsin; CRP: C-reactive protein.

**Table 4:** Characteristics of patients, according to severity of COVID-19.

n = 2217	Non-Severe COVID-19 n = 1145 (56.8)	Severe COVID-19 n = 872 (43.2)	*p*-Value
Age, years, m (SD)	58.9 (14.7)	62.2 (12.8)	<0.001
Gender (male), n %	54.3	67.4	<0.001
Smoking status, n (%)			
▪ Current smoker	83 (7.3)	37 (4.3)	0.005
▪ Former smoker	393 (34.7)	400 (46.1)	<0.001
IPA, median (IQR)	20 (10–40)	25 (15-40)	<0.001
Body mass index, kg/m^2^, m (SD)	29 (25.7–33.3)	29.4 (26.7–32.6)	0.140
Pulmonary comorbidity, n (%)	243 (21.2)	183 (21)	0.897
▪ COPD, n (%)	65 (5.7)	57 (6.5)	0.418
▪ Asthma, n (%)	117 (10.2)	69 (7.9)	0.076
▪ ILD	13 (1.1)	10 (1.2)	0.978
Home oxygen therapy, n (%)	9 (0.8)	15 (1.7)	0.055
▪ Comorbidities, n (%)			
Diabetes_mellitus	174 (15.2)	210 (24.1)	<0.001
Hypertension	402 (35.1)	391 (44.8)	<0.001
Dyslipemia	301 (26.3)	374 (42.9)	<0.001
Coronary artery disease	98 (8.6)	127 (14.6)	<0.001
Nephropathy	45 (3.9)	45 (5.2)	0.188
Hepatopathy	39 (3.4)	41 (4.8)	0.135
Immunosuppression	34 (3)	29 (3.3)	0.649
History of cancer	72 (6.3)	75 (8.6)	0.048
AAT mg/dL, m (SD)	131.9 (31.1)	132.2 (26.6)	
▪ ≥116, n (%)	704 (76.9)	544 (70.5)	0.859
▪ <116 y ≥ 60, n (%)	209 (22.8)	223 (28.9)	0.006
▪ <60, n (%)	2 (0.2)	5 (0.6)	
A1AT mg/dL, %			0.003
▪ Level ≥ 116, n (%)	704 (76.9)	544 (70.5)	
▪ Level < 116, n (%)	211 (18.4)	228 (29.5)	
A1AT genotyping test, n (%)			0.022
▪ Absence mutations	892 (81.2)	656 (77)	
▪ Presence mutations (%)	206 (18.8)	196 (23)	
MI, n (%)	3 (0.3)	3 (0.4)	
MS, n (%)	166 (15.1)	150 (17.6)	
MZ, n (%)	14 (1.3)	27 (3.2)	
MM malton	2 (0.2)	0	
MP lowell	4 (0.4)	1 (0.1)	
MM procida	1 (0.1)	0	
SS	13 (1.2)	9 (1.1)	
SZ	2 (0.2)	3 (0.4)	
ZZ	1 (0.1)	3 (0.4)	

Abbreviations: IPA: pack-years; COPD: chronic obstructive pulmonary disease, ILD: diffuse interstitial lung disease, AOS: sleep apnea syndrome, ICU: intensive care unit, UCRI: intermediate respiratory care unit; NIV/CPAP: non-invasive ventilation/continuous positive airway pressure; A1AT: alpha-1 antitrypsin; CRP: C-reactive protein.

**Table 5:** Clinical associations with a severe COVID-19 disease.

	OR (95% CI)	*p*-Value
Age	1.017 (1.011–1.024)	<0.001
Gender (female)	0.573 (0.477–0.689)	<0.001
Diabetes_mellitus	1.773 (1.417–2.218)	<0.001
Hypertension	1.500 (1.253–1.797)	<0.001
Dyslipemia	2.105 (1.744–2.540)	<0.001
Coronary artery disease	1.820 (1.376–2.408)	<0.001
History of cancer	1.401 (1.001–1.961)	0.049
Current smoker	0.568 (0.381–0.846)	0.005
Former smoker	1.611 (1.344–1.930)	<0.001
A1AT level < 116 mg/dL	1.398 (1.124–1.739)	0.003
Presence mutations	1.294 (1.038–1.612)	0.022

## Data Availability

The datasets used and/or analyzed during the current study are available from the corresponding author on reasonable request.

## Abbreviations

AATD = alpha-1 antitrypsin deficiency; A1AT = alpha-1 antitrypsin; SARS-CoV-2 = severe acute respiratory syndrome coronavirus 2; COVID-19 = coronavirus disease 2019; IPA: Pack-years; COPD: chronic obstructive pulmonary disease, ILD: diffuse interstitial lung disease, AOS: sleep apnea syndrome, ICU: intensive care unit, UCRI: intermediate respiratory care unit; HFNC = high-flow nasal cannula; NIV/CPAP: non-invasive ventilation/continuous positive airway pressure; CRP: C-reactive protein. IQR = interquartile range.
